# Epigenetic priming of an epithelial enhancer by p63 and CTCF controls expression of a skin-restricted gene *XP33*

**DOI:** 10.1038/s41420-023-01716-3

**Published:** 2023-12-08

**Authors:** Artem Smirnov, Anna Maria Lena, Giulia Tosetti, Xue Yang, Angela Cappello, Manuela Helmer Citterich, Gerry Melino, Eleonora Candi

**Affiliations:** 1 Department of Experimental Medicine, Torvergata Oncoscience Research, University of Rome “Tor Vergata”, via Montpellier 1, 00133 Rome, Italy; 2 Biochemistry Laboratory, Istituto Dermopatico Immacolata (IDI-IRCCS), 00166 Rome, Italy; 3 The Third Affiliated Hospital of Soochow University, Institutes for Translational Medicine, Soochow University, 215000 Suzhou, China; 4 Interdisciplinary Department of Medicine University of Bari “Aldo Moro”, 70124 Bari, Italy; 5 Biology Department, University of Rome “Tor Vergata”, Via della Ricerca Scientifica, snc, 00133 Rome, Italy

**Keywords:** Chromatin remodelling, Transcriptional regulatory elements

## Abstract

The transcription factor p63 is a renowned master regulator of gene expression of stratified epithelia. While multiple proteins have been identified as p63 bona fide targets, little is known about non-coding RNAs (ncRNAs) whose transcription is controlled by p63. Here, we describe a skin-specific non-coding RNA *XP33* as a novel target of p63. *XP33* levels are increased during keratinocyte differentiation in vitro, while its depletion results in decreased expression of late cornified gene *LCE2D*. By using publicly available multi-omics data, we show that CTCF and p63 establish an epithelial enhancer to prime *XP33* transcription in a tissue-restricted manner. *XP33* promoter and enhancer form a chromatin loop exclusively in keratinocytes but not in other cell types. Moreover, the *XP33* enhancer is occupied by differentiation-specific factors that control *XP33* transcription. Altogether, we identify a tissue-specific non-coding RNA whose expression is epigenetically regulated by p63 and CTCF.

## Introduction

Being the largest organ, the skin protects the human body from physical, mechanical and chemical traumas, as well as prevents it from infection and dehydration. Starting from single epidermal stem cells, human keratinocytes proliferate within a basal layer and then differentiate into specialised cells which move upwards. The dead cells of the upper layers of the epidermis form so-called cornified envelope harbouring a plethora of hydrophobic protein complexes which allow the skin to function as a barrier [1].

Epidermal differentiation is an extremely complex and exquisitely orchestrated process involving a myriad of players at epigenetic, transcriptional, protein [2] and metabolic [3] levels. Perturbation of the differentiation programme in epidermis or other stratified epithelia leads to the development of pathologies. Delay of cellular differentiation or dedifferentiation is also considered a hallmark of cancers [4–6]. For instance, in head and neck squamous cell carcinoma aberrations of genes regulating proliferation and squamous differentiation contribute to squamous cell carcinogenesis [7–13]. In vitro, induction of keratinocyte differentiation leads to dramatic epigenetic changes and reorganisation of chromatin structure. Early attempts to describe human transcriptome revealed the existence of a plethora of non-coding RNAs (ncRNAs) of unknown function. In recent years, lncRNAs have been shown to allow a fine-tuning of complex tissue- and spatiotemporal processes within the human body such as development or response to external stimuli. In fact, many ncRNAs are expressed at low levels only in determined temporal windows within specific cell types. ncRNA is also deregulated in epithelial diseases, including epithelial tumours [14–20]. ncRNA are found both in the nucleus and cytoplasm and are involved in a broad range of interactions with protein complexes and chromatin. Importantly, we and others have described several long ncRNAs (lncRNAs) including uc.291 [21, 22] and TINCR [23] implemented in terminal differentiation of keratinocytes by directly interacting with chromatin remodellers.

p63 is a master gene regulating the fate of epithelial cells [24–27] and a few other tissues such as the ovary [28]. Being a transcription factor, p63 controls the expression of lineage-specific genes and directs multiple processes including proliferation and differentiation of epithelial cells. Genome-wide studies of the p63 binding landscape unveiled its essential role in establishing lineage-restricted enhancers [29]. p63 interacts with chromatin modellers to increase chromatin accessibility at epithelial enhancers. Moreover, p63 co-operates with insulator CTCF which enables the formation of three-dimensional loops between lineage-specific enhancers and promoters of target genes [30]. While multiple proteins involved in epidermal differentiation have been described as p63 targets genes, little is known about lncRNAs regulated by this transcription factor.

Here, we analyse the expression of lncRNAs in differentiating keratinocytes lacking p63. We identify *XP33*, a skin-specific lncRNA induced at the late stages of epidermal differentiation in vitro. *XP33* expression is regulated by an epithelial enhancer located downstream *LCE2* locus. We use publicly available multi-omics data and show that this enhancer is constitutively bound by CTCF but primed by p63 only in epithelial cells. We show that the *XP33* promoter and LCE2 enhancer form a loop in keratinocytes but not in other types of epithelial cells, possibly maintained by epithelial transcription factor ZNF750, KLF4 and EGR3.

## Results

### Expression of skin-specific ncRNA *XP33* is affected by p63 depletion

**Fig. 1:**
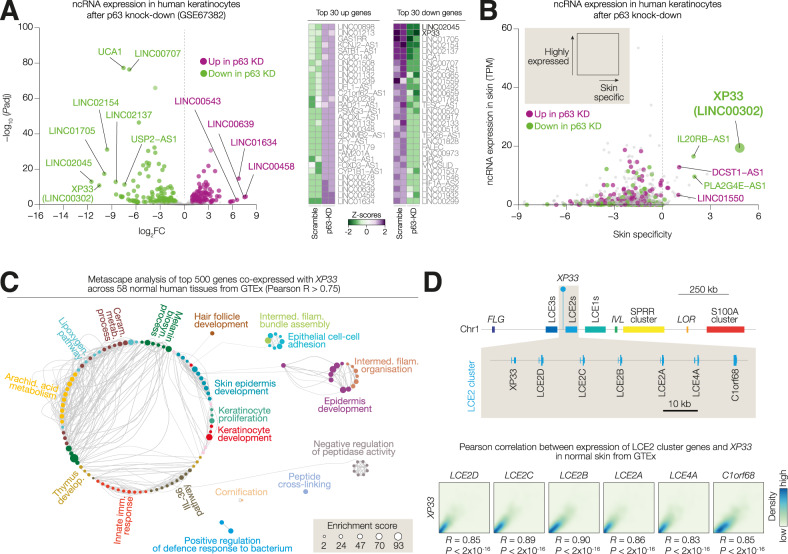
Expression of skin-specific ncRNA *XP33* is affected by p63 depletion. **A** Volcano plot showing modulated ncRNAs after p63 knock-down in differentiated keratinocytes. The top 30 down- and upregulated ncRNAs are shown in a heatmap on the right. **B** Dot plot showing the distribution of the ncRNAs from (A) based on the expression in the skin (in TPM) and skin-specificity score. **C** Metascape analysis of the top 500 genes co-expressed with *XP33* across human tissues from GTEx. **D** (Top) Schematic showing the genomic location of *XP33* within the LCE2 locus on human Chromosome 1. (Bottom) Correlation plots show a correlation between expression levels of *XP33* and levels of genes from the LCE2 locus in human skin.

In order to identify lncRNAs differentially expressed in keratinocytes lacking p63, we retrieved lncRNA gene expression values from publicly available RNA sequencing data [31] carried out in differentiated keratinocytes after p63 knock-down (Fig. 1A). We identified 125 down- and 134 upregulated lncRNAs in p63-KD cells compared to scramble control (abs(logFC)>1 and *P* < 0.05). Of note, we observed a stronger significant down-regulation of lncRNA expression (up to log(FC) = −12) with *LINC02045* and *XP33* (*LINC00302*) being the most down-regulated lncRNAs (Fig. 1A, heatmap). We then assessed the abundance of that lncRNA in the skin as well as the specificity of expression in the skin by analysing their expression in the skin versus other human tissues in GTEx (Fig. 1B and Table S3). *XP33* was the most skin-specific (skin-specificity score >5) lncRNA expressed in skin (expression >10 TPM) p63-regulated gene. Accordingly, *XP33* expression was barely detectable in any tissues other than the skin (Supplementary Fig. S1A). To get insights into the *XP33* expression pattern, we identified 500 top genes co-expressed with *XP33* by calculating the Pearson correlation coefficient (R) between *XP33* expression and the expression of ~57.000 genes across 58 human tissues from GTEx. Metascape analysis revealed that these genes mainly belong to epidermis development pathways (Fig. 1C). *XP33* gene is located on chromosome 1 within the epidermal differential complex, containing several clusters of genes essential for terminal differentiation of keratinocytes (Fig. 1D). Due to *XP33* location adjacent to *LCE2D* gene, we hypothesised that *XP33* was part of the *LCE2* cluster. In fact, its expression is highly correlated (*R* > 0.85) with an expression of other genes within the *LCE2* cluster (*LCE2A/B/C/D, LCE4A* and *C1orf68*) (Fig. 1D).

Collectively, we identify *XP33* as skin-specific lncRNA whose expression is decreased after p63 knock-down.

### *XP33* expression is upregulated during induced keratinocyte differentiation

**Fig. 2:**
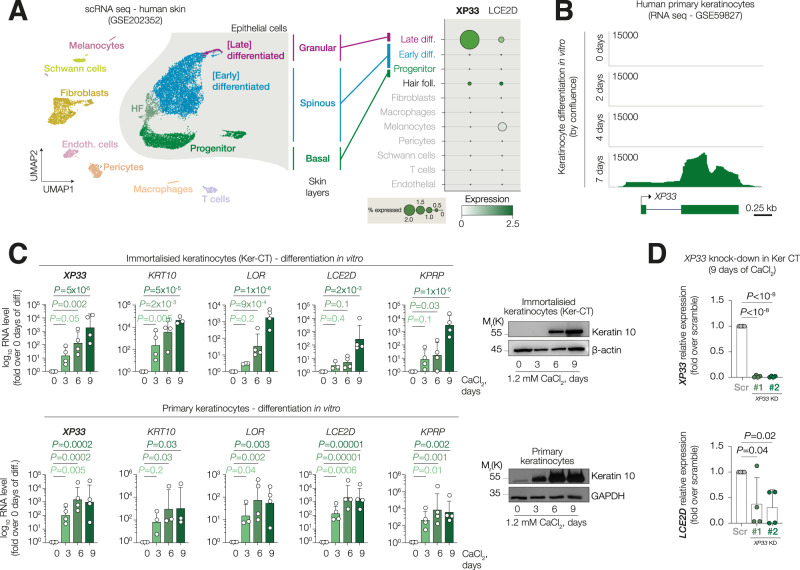
*XP33* expression is upregulated during induced keratinocyte differentiation. **A** (Left) UMAP plot showing clusters of cells from human skin. (Right) Dot plot showing expression of *XP33* and *LCE2D* in identified clusters. **B** RNA-seq signal tracks showing XP33 expression in human keratinocytes induced for differentiation for 2, 4 or 7 days. **C** qPCR analysis of *XP33*, *KRT10*, *LCE2D*, *LOR*, and *KPRP* expression in immortalised cells Ker-CT (top) or primary keratinocytes (bottom). Western blot confirms induction of differentiation by Keratin 10 staining. *n* = 4 (biological replicates), *P* by one-way ANOVA. **D** qPCR analysis of *XP33* and *LCE2D* expression in Ker-CT silenced for *XP33* and induced to differentiate for 9 days. *n* = 4 (biological replicates), *P* by one-way ANOVA.

Bulk RNA-seq analysis of gene expression in skin from GTEx only shows average expression of the whole tissue, therefore, to understand which types of skin cells express *XP33*, we performed de novo analysis of previously published scRNA-seq data (Fig. 2A). By clustering cells using UMAP and annotating clusters with SingleR packages, we were able to detect major epithelial and non-epithelial cell types. *XP33* was detectable only in a handful of cells from late differentiation cluster corresponding to upper-spinous and granular layers of the epidermis, while its expression was hardly detectable in progenitor keratinocytes or any other non-epithelial cells in contrast to previous report [32]. Of note, the *XP33* expression pattern was similar to its genomic neighbour *LCE2D* (Fig. 2A). To investigate *XP33* behaviour during keratinocyte differentiation in vitro, we analysed *XP33* levels in a publicly available RNA sequencing of confluence-induced differentiation of primary human keratinocytes and we saw an up-regulation of this transcript at 7 days of differentiation while in progenitor cells it was barely detectable (Fig. 2B). We then confirmed this observation carrying out the differentiation of both primary and immortalised keratinocytes (Ker-CT) induced by treatment with 1.2 mM calcium chloride (Fig. 2C). Accordingly, *XP33* levels increased significantly after 6 days of differentiation (up to 1000-fold) similar to late cornified genes *LOR*, *LCE2D* and *KPRP*, while expression of an early marker keratin 10 was strongly upregulated already after 3 days of calcium treatment. *XP33* role has been previously discussed in dermal fibroblasts [32], however, it remains unclear in keratinocytes. Therefore, we knocked-down *XP33* expression in Ker-CT cells using two distinct small-interfering RNAs (siRNAs) and induced the cells to differentiate for 9 days (Fig. 2D). Since many lncRNAs are known to regulate the expression of nearby genes, we assessed the expression of its neighbour *LCE2D*. Interestingly, after *XP33* knock-down, *LCE2D* levels were significantly reduced.

Altogether, these data demonstrate that *XP33* is expressed during the late stages of epidermal differentiation and it may function to promote the expression of neighbouring genes like *LCE2D*.

### An epithelia-specific enhancer within the LCE2 locus is activated during differentiation

**Fig. 3:**
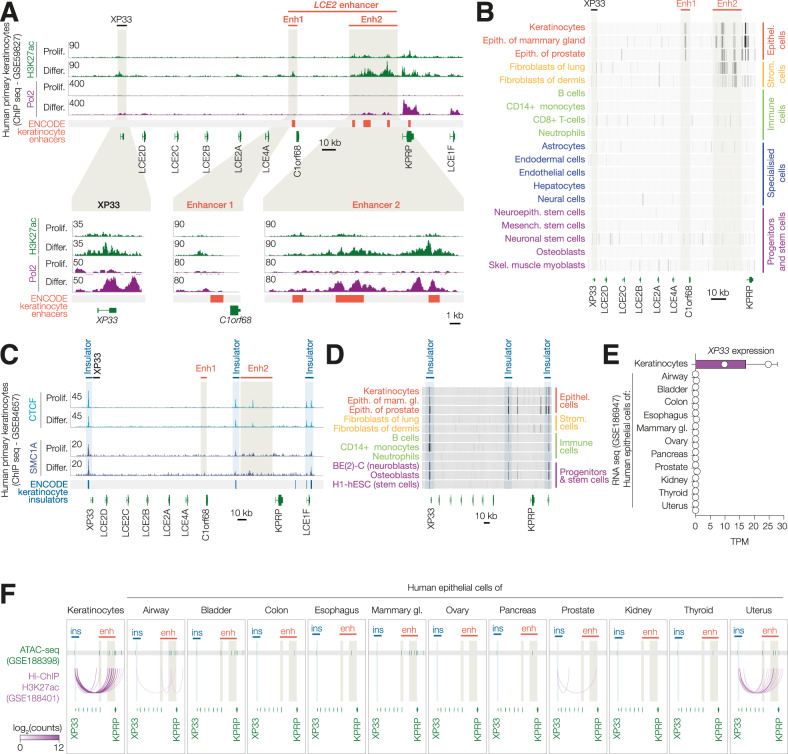
An epithelia-specific enhancer within the LCE2 locus is activated during differentiation. **A** ChIP-seq tracks of H3K27ac and Pol2 in keratinocytes (proliferating or differentiated). Enlarged areas of *XP33* promoter, Enhacer 1 and Enhacer 2 are shown below. **B** Dense ChIP-seq tracks of H3K27ac in different cell types from ENCODE. **C** ChIP-seq tracks of CTCF and SMC1A in keratinocytes (proliferating or differentiated). **D** Dense ChIP-seq tracks of CTCF in different cell types from ENCODE. **E** *XP33* expression from RNA-seq in cells derived from different types of epithelia. **F** ATAC-seq regions (top) and H3K27ac-HiChIP chromatin loops (bottom) in the same samples.

To study the regulation of *XP33* expression, we analysed the chromatin state of the *LCE2* locus on chromosome 1 using publicly available ChIP-sequencing data (Fig. 3A). We observed an increase in H3K27 acetylation at *XP33* and *C1orf68* promoters accompanied by an increase of Pol2 occupancy in differentiated keratinocytes, which is consistent with the notion that *XP33* is only expressed during late stages of differentiation. Furthermore, a region adjacent to the *C1orf68* gene and a large region between *C1orf68* and *KRPR* genes had multiple sites of H3K27ac present already in progenitor cells with a further increase in differentiated keratinocytes. Both regions overlapped with predicted ENCODE keratinocyte enhancers (hereinafter, *LCE2* enhancer). Of note, we were able to detect an increase of Pol2 occupancy at *LCE2* enhancer in differentiated cells. Analysis of ENCODE ChIP-seq data across multiple tissues revealed the presence of the H3K27ac mark within the *LCE2* enhancer region only in epithelial cells of the skin, mammary gland and prostate, but not in other types of cells including immune cells, soft tissue cells or stem cells (Fig. 3B). This suggests that *LCE2* enhancer is epithelia-specific.

It has been established that lineage-specific enhancers can regulate the expression of specialised clusters of genes by physically interacting with their promoters [33]. Distal regulatory elements can interact via the formation of chromatin loops maintained by an insulator protein CTCF and cohesin complex. We investigated CTCF occupancy in human keratinocytes and found two distinct sites bound by CTCF flanking *LCE2* enhancer and one CTCF site upstream *XP33*. Furthermore, these regions were also occupied by the SMC1A subunit of the cohesin complex (Fig. 3C). CTCF and cohesin occupancy did not change during differentiation. In contrast to the H3K27ac mark, we saw CTCF binding at identified insulator regions in virtually all cell types analysed, including human embryonic stem cells H1-hESC (Fig. 3D).

To further investigate a three-dimensional structure of *XP33-LCE2* cluster in keratinocytes, we simultaneously assessed the expression of *XP33*, chromatin accessibility and chromatin looping in *LCE2* locus across 15 types of epithelial cells including keratinocytes using publicly available data from a recent study [34]. As expected, *XP33* was found expressed only in keratinocytes but not in any other epithelial cell types analysed (Fig. 3E). Interestingly, *LCE2* enhancer was accessible in a few types of cells, including lung, bladder, mammary gland epithelial cells similar to keratinocytes (Fig. 3F, the upper part—ATAC-seq). Nonetheless, H3K27ac-HiChIP revealed *XP33*-enhancer looping only in keratinocytes and to a lesser extent in epithelial cells of the uterus, but not other tissues (Fig. 3F, lower part—HiChIP-seq). These findings might explain why *XP33* expression is restricted to keratinocytes, while *LCE2* enhancer is active and accessible in multiple epithelial cell types.

Collectively, our data suggest that *XP33* expression is regulated via promoter-enhancer interaction which is specific to keratinocytes.

### p63 is a pioneer factor essential for the establishment of *LCE2* enhancer

**Fig. 4:**
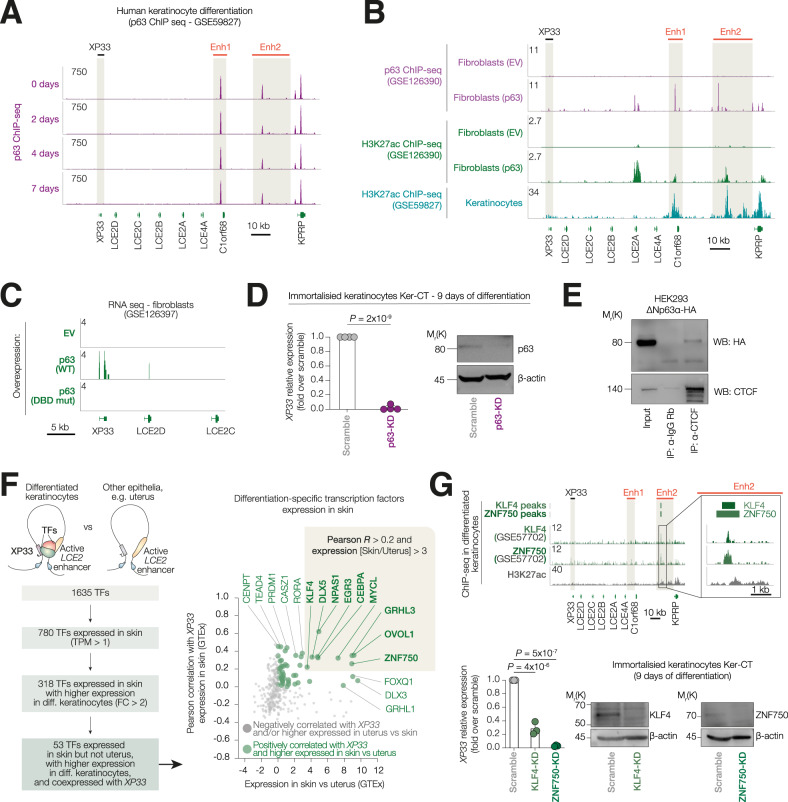
p63 is a pioneer factor essential for the establishment of an *LCE2* enhancer. **A** ChIP-seq signal tracks for p63 in human keratinocytes induced for 2, 4 or 7 days of differentiation. **B** ChIP-seq signal tracks for p63 in H3K27ac in human fibroblasts expressing either empty vector or ΔNp63α. At the bottom, ChIP-seq signal tracks for H3K27ac in human fibroblasts keratinocytes. **C** RNA-seq signal track of fibroblast expressing either empty vector, or wild-type ΔNp63α, or DND-binding domain mutant of ΔNp63α. **D** qPCR analysis of *XP33* expression in Ker-CT was silenced for p63 and induced to differentiate for 9 days. Western blot confirms an efficient knock-down. *n* = 4 (biological replicates), *P* by one-way ANOVA. **E** Co-immunoprecipitation in HEK293 overexpressing ΔNp63α-HA using either anti-CTCF or anti-IgG antibodies. Western blot for HA and CTCF. *n* = 2 (biological replicates). **F** (Left) Schematic shows strategy to select differentiation-specific skin-restricted TFs which may regulate the expression of *XP33*. (Right) Dot plot showing the distribution of human TFs based on the specificity of expression in skin and correlation of their expression with levels of *XP33*. Top hits are shown in a beige box. **G** (Top) ChIP-seq signals for KLF4 and ZNF750 in differentiated keratinocytes. The enlarged area of the LCE2 locus is shown on the right. (Bottom) qPCR analysis of *XP33* expression in Ker-CT silenced for KLF4 or ZNF750 and induced to differentiate for 9 days. Western blot confirms an efficient knock-down. *n* = 3 (biological replicates), *P* by one-way ANOVA.

Since we identified *XP33* as a p63-regulated gene and considered a pivotal role of p63 in the establishment of epidermal enhancers, we questioned whether the *LCE2* enhancer was occupied by p63 (Fig. 4A). We found several sites bound by p63 within *LCE2* enhancer, both at enhancer 1 (adjacent to *C1orf68*) and large enhancer 2 (between *C1orf68* and *KPRP*). p63 binding to *LCE2* enhancer did not change during differentiation suggesting that p63 could epigenetically regulate this region already in progenitor cells.

To understand whether p63 is essential for *LCE2* enhancer activation, we investigated both p63 and H3K27ac occupancy in fibroblasts analysing data from a recent study [29] (Fig. 4B). Exogenous p63 bound the same loci in fibroblasts as endogenous p63 in keratinocytes. Moreover, the same loci gained H3K27 acetylation after p63 overexpression. Interestingly, only overexpression of wild-type p63 but not DNA-binding domain mutant of p63, led to a mild activation of *XP33* expression in fibroblasts (Fig. 4C). We then analysed impact of p63 loss on *XP33* expression in vitro (Fig. 4D). We observed a significant decrease of *XP33* expression in differentiated (9 days) keratinocytes Ker-CT after p63 knock-down. As the *LCE2* locus is occupied by CTCF in multiple cell types of non-epithelial origin, we questioned whether p63 could use CTCF as a bait to recognise enhancer regions. p63 was shown to co-operate with CTCF to establish epithelia-specific chromatin architecture [30], even though physical interaction between the two proteins has not been investigated. We, therefore, performed a co-immunoprecipitation of CTCF in HEK293 cell overexpressing ΔNp63α-HA and were able to detect interaction between CTCF and p63.

Since *XP33* is specifically expressed in differentiated keratinocytes but not in other epithelial or progenitor cells harbouring an active *LCE2* enhancer, we questioned whether there were any specific transcriptional factors (TFs) able to confer *XP33* expression in a lineage-specific fashion (Fig. 4F). To address this question, we screened a list of 1635 human TFs based on their expression in skin and a trend for a higher expression in differentiated keratinocytes. We selected 318 TFs and then further screened them according to a co-expression with *XP33* in skin (by Pearson correlation) and specificity of expression in the skin over other epithelia harbouring active *LCE2* enhancers (e.g., uterus). We identified 9 candidate TFs that could potentially regulate *XP33* expression (Fig. 4F). Of note, EGR3 has been already shown to bind LCE2 enhancers [35]. We, therefore, analysed the occupancy of two major TFs, ZNF750 and KLF4, essential for keratinocyte differentiation [36]. In fact, ZNF750 and KLF4 bound a large portion of *LCE2* enhancer (Fig. 4G) and their expression was positively correlated with XP33 expression in human skin samples. Furthermore, ZNF750 and KLF4 knock-down in Ker-CT cells resulted in significantly reduced levels of *XP33*.

Altogether, our analyses show that p63 is a pioneer factor that establishes lineage-specific *LCE2* enhancer, while differentiation-specific TFs, like EGR3, ZNF750 and KLF4, may drive *XP33* expression in a tissue-restricted manner.

## Discussion

Here, we analyse the expression of ncRNAs in differentiated keratinocytes depleted for p63 and identify over 300 modulated non-coding genes. However. most of those genes are expressed at very low levels in the skin or show a higher expression in other tissues, potentially leading to the identification of physiologically irrelevant genes. Given the importance of p63 for epidermal homoeostasis, we only focused our study on p63-regulated ncRNAs specific to the skin. Thus, we identified *XP33*, also known as long intergenic non-coding RNA 302 (LINC00302), as a skin-restricted and the most down-regulated gene in p63-KD cells. *XP33* gene is located on chromosome 1 within the epidermal differentiation complex adjacent to *LCE2D* gene [37]. *XP33* expression in skin is highly correlated with the expression of genes from the *LCE2* locus, moreover, the most co-expressed genes for *XP33* across human tissues are related to epidermal differentiation pathways. By analysing both primary and immortalised keratinocytes, we showed that *XP33* levels drastically increase during in vitro calcium chloride-induced differentiation. Furthermore, single-cell analysis revealed that *XP33* expression is restricted to upper-spinous and granular layers of skin. The same trend was observed during confluence-induced differentiation as shown by analysing a previously published RNA-seq. All these observations are in line with the notion that *XP33* is a ncNRA related to the terminal differentiation of keratinocytes.

**Fig. 5:**
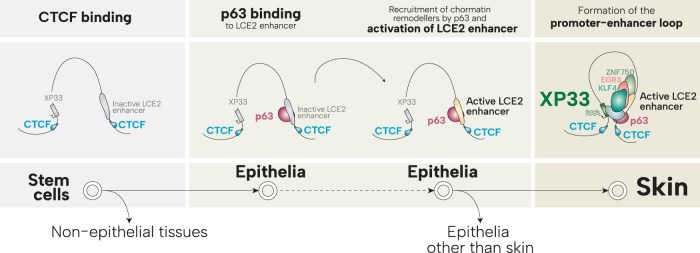
p63 and CTCF prime an epithelial enhancer to boost expression of XP33. CTCF binds insulator sites upstream XP33 promoter and within LCE2 enhancer in multiple cells including stem cells. However, only in epithelial cells, p63 activates LCE2 enhancer. Active LCE2 enhancer interacts with XP33 promoter only in differentiating keratinocytes leading to its expression controlled by ZNF750, KLF4 and EGR3.

We characterised the chromatin state of the region surrounding the *XP33* gene by analysing publicly available ChIP-seq experiments carried out in human keratinocytes. We found two loci resembling active enhancers downstream of *XP33* (collectedly called as *LCE2* enhancer). Interestingly, *LCE2* enhancer shows high levels of H3K27 acetylation in keratinocytes, mammary gland and prostate epithelial cells, but not in other specialised cells, stromal, immune or stem cells. This suggests the presence of an active *LCE2* enhancer in distinct epithelia. Nonetheless, *XP33* expression is restricted to the skin. This might be explained by the fact that active *LCE2* enhancer physically interacts with *XP33* region only in keratinocytes but not in other epithelial as shown by analysis of a HiChIP experiment. However, these findings must be confirmed by Hi-C or conventional chromatin conformation capture experiments. Mechanistically, interaction between regulatory elements of chromatin is mediated by an insulator factor CTCF and cohesin complex which establish and maintain three-dimensional chromatin loops. We observed strong CTCF binding both upstream *XP33* and within regions flanking *LCE2* enhancer. The same sites were co-bound by SMC1A, a component of the cohesin complex. CTCF binding remained unchanged during keratinocyte differentiation in vitro. Furthermore, CTCF binding upstream *XP33* promoter was found in multiple cell types, including epithelia, immune, stromal and stem cells. This may suggest that CTCF marks the boundaries of *XP33*-enhancer loop early during development (Fig. 5), however, due to the closed chromatin within the enhancer region and the absence of specific TFs, this loop remains unstable in most of the cell types.

Similar to CTCF, we found p63 to bind several loci within the *LCE2* enhancer and importantly this binding was unchanged during differentiation. It has been speculated that p63 is a pioneer factor that promotes the establishment of epithelial enhancers via direct binding to its motifs on inaccessible chromatin and recruitment of chromatin remodellers. In fact, we observed that overexpression of p63 in stromal cells which normally have *LCE2* enhancer in inactive form, led to an increased acetylation of H3K27 within this region (Fig. 5). Moreover, overexpression of p63 in fibroblast was able to induce mild levels of *XP33* expression. We demonstrated a physical interaction between p63 and CTCF in vitro, therefore, it is plausible that p63 co-operates with CTCF to establish *LCE2* enhancer / *XP33* promoter interaction [30]. Undoubtedly, further in vitro experiments are needed to investigate whether loss of p63 or CTCF in keratinocytes leads to closed chromatin conformation of the *LCE2* enhancer.

p63 activity in proliferating keratinocytes is necessary to enable *XP33* expression during differentiation. Our knock-down experiments confirmed that depletion of p63 led to a dramatic decrease of *XP33* at 9 days of differentiation. However, since p63 binding to *LCE2* enhancer did not change during differentiation in vitro, it is unlikely that p63 is sufficient to drive *XP33* expression during terminal differentiation of keratinocytes. Moreover, in human skin p63 expression is gradually decreased with barely detectable levels of p63 in late spinous layers of the epidermis. One possibility could be that p63 is essential to pre-mark *LCE2* enhancer which remains active even when p63 expression begins to decrease in differentiating cells. We hypothesised that other transcription factors, specific to differentiated keratinocytes, might bind *LCE2* enhancers to induce *XP33* expression during terminal stages of epidermal specialisation. We have screened >1600 human TFs based on their expression in skin, their trend towards an increased expression in differentiated cells, their co-expression with *XP33* and skin-restricted expression. We found nine TFs that potentially may regulate *XP33* expression. Of note, EGR3 binding to this region has been already described previously [35]. We also found ZNF750 and KLF4, two TFs known to regulate terminal differentiation [36, 38, 39], to bind *LCE2* enhancers. Knock-down of both factors significantly reduced levels of *XP33* in differentiated cells, suggesting their involvement in the transcription of *XP33* and possibly other *LCE2* genes (Fig. 5).

Little is known about the function of this lncRNA. *XP33* was found to be differentially expressed in psoriasis [40], although its role remains unclear. Our knock-down experiments showed that depletion of *XP33* resulted in decreased expression of its genomic neighbour *LCE2D* which may suggest a cis-acting mechanism of gene expression regulated by *XP33*, however, this must be investigated more in detail.

In summary, we characterise the lncRNA *XP33* and lineage-restricted regulation of its expression. Further work will be needed to understand the function of *XP33* in human keratinocytes, epidermal differentiation, and in pathological conditions with loss of differentiated phenotype. Our study also provides a set of other p63-regulated lncRNAs which can be further investigated.

## Materials and methods

### Cell culture and treatments

Human epidermal keratinocytes (neonatal) were purchased from Lonza (#00192907). Immortalised human keratinocytes Ker-CT were purchased from ATCC (#CRL-4048, RRID:CVCL_S877). Keratinocytes were grown in sub-confluent conditions in EpiLife medium (ThermoFisher, #MEPI500CA) supplemented with growth factors (ThermoFiscer, #S0015) on dishes pre-coated with rat tail collagen type I solution (Corning, #354236). HEK293 cells were grown in Dulbecco’s modified Eagle’s medium (ThermoFisher) supplemented with 10% (vol/vol) fetal bovine serum (ThermoFisher) and 1% penicillin/streptomycin (ThermoFisher). To induce differentiation, keratinocytes were seeded at high confluence and treated with 1.2 mM calcium chloride added directly into the growth medium for indicated times.

### RNA interference

RNA interference experiments were performed using Lipofectamine RNAiMAX (ThemoFisher, 13778075) following the manufacturer’s instructions. Cells were seeded onto 12-well plates and transfected with 50 pmol of siRNA and 2.5 µL of the transfection reagent. The day after the first transfection, the cells were induced to differentiate as described before. The transfection was repeated on day 5 of differentiation. Cells were collected at day 9 of differentiation. All siRNAs were purchased from Sigma. siRNA sequences are listed in the Table S1. KLF4 and ZNF750 siRNA sequences were obtained from [41], while TP63 siRNA sequences were obtained from [42]. KLF4 and ZNF750 siRNAs were used as a pool of two siRNAs.

### RNA extraction and reverse transcription-quantitative polymerase chain reaction (RT-qPCR)

Total RNA was extracted using an RNeasy mini kit (Qiagen, #74004) with DNase I treatment step (Qiagen, #79254) following the manufacturer’s instructions. One microgram of total RNA was used for retrotranscription with SensiFAST cDNA Synthesis Kit (BioLine, # BIO-65054). qPCR on cDNA was performed using PowerUp SYBR Green Master Mix (ThermoFisher, #A25778) in QuantStudio 5 Real-Time PCR System. All samples were run in triplicate. *GAPDH* was used as house-keeping gene. Differences in gene expression were calculated using the ΔΔCt method. Sequences of the qPCR primers are listed in the Table S1.

### Western blot

Keratinocytes were lysed directly on the dish with SDS buffer (100 mM Tris рН 8.8, 1% SDS, 5 mM EDTA, 20 mM DTT, and 2 mM AEBSF). Lysates were resolved in SDS polyacrylamide gel and blotted onto an Amersham Hybond PVDF membrane (GE Healthcare, #GE10600023). Membranes were incubated with primary antibodies overnight at 4 °C. The day after the membranes for washed with 0.1% PBS/Tween-20 three times and incubated with secondary antibodies for 1 h at room temperature. The membranes were washed three times. The signal was revealed using Western Lightning Plus Chemiluminescent Substrate (Revvity, # NEL103E001EA) in the Uvitec Alliance imaging system (Uvitec) or by exposure of X-ray films. The following primary antibodies were used: anti-Keratin-10 (1:1000, BioLegend, #905403, RRID:AB_2749902), anti-GAPDH (1:15000, Sigma, # G8795, RRID:AB_1078991), anti-bet-actin (1:10.000, Sigma, #A5441, RRID:AB_476744), anti-p63alpha (1:500, D2K8X, Cell Signaling, #13109, RRID:AB_2637091), anti-CTCF (D31H2 XP, Cell signaling, #3418, RRID:AB_2086791), anti-HA (BioLegend, #901513, RRID:AB_2565335), anti-KLF4 (R&D systems, AF3640, RRID:AB_2130224), anti-ZNF750 (Sigma, HPA023012-100ul, RRID:AB_1859439), goat anti-mouse IgG (H + L)-HRP conjugate (1:10.000, BioRad, #1706516, RRID:AB_11125547), goat anti-rabbit IgG (H + L)-HRP conjugate (1:10.000, BioRad, #1706515, RRID:AB_11125142), and rabbit anti-rabbit IgG (H + L)-HRP conjugate (1:10.000, BioRad, #1706515, RRID:AB_11125142). Uncropped western blots are shown in Supplementary Fig. S1B.

### Co-immunoprecipitation (co-IP)

HEK293 cells were transfected with pcDNA3.1-HA (empty vector, “EV”) or pcDNA3.1-ΔNp63α-HA expressing vectors using Effectene Reagent (Qiagen, Hilden, Germany) according to the manufacturer’s protocol. Twenty-four hours after transfection cells were lysed in triton buffer (NaCl 150 mM, Tris HCl pH [5, 7] 50 mM, Triton 0,5%, NaF 50 mM, EDTA [pH 8] 1 mM, 0.1 mM Sodium orthovanadate and cOmplete EDTA-free protease inhibitor cocktail (Merck, #11873580001)). Total protein extract was quantified and 2 mg was used for each co-IP. Lysate pre-clearing was performed by 2 h incubation at 4 °C with 25 µL of Protein A Sepharose 4 Fast Flow (Cytiva, #17528001) diluted in 1 mL of Triton buffer. Two microgram of anti-CTCF (D31H2 XP, Cell signaling, #3418, RRID:AB_2086791) or anti-rabbit IgG isotype Control (Invitrogen, #10500 C, RRID:AB_2532981) antibodies were coated to 25 µL of Protein A Sepharose 4 Fast Flow (Cytiva, #17528001, RRID:AB_2532981) by 2 h incubation at 4 °C in 1 mL of Triton buffer. Co-IP was carried out by overnight incubation of precleared lysates with antibody-coated beads at 4 °C. The day after, beads were washed 6 times in 1 mL of triton buffer, then boiled for 10 min at 98 °C in 2 × 40 µL of Laemmli buffer and analysed by WB.

### Publicly available multi-omics data

All the data used in the study are available on GEO NCBI: RNA-seq of human keratinocytes after p63 knock-down (GSE67382) [31], scRNA-seq of human skin (GSE202352) [43], RNA-seq and ChIP-seq for H3K27ac, p63, and Pol2 in human keratinocytes differentiated in vitro (GSE59827) [44], ChIP-seq for CTCF and SMC1A in differentiated human keratinocytes (GSE84657) [33], RNA-seq, ATAC-seq and H3K27ac-HiChIP of epithelia (GSE188405) [34], RNA-seq and ChIP-seq for H3K27ac and p63 in human fibroblasts (GSE126390) [29], ChIP-seq for ZNF750 and KLF4 in differentiated human keratinocytes (GSE57702) [36]. ChIP-seq for H3K27ac and CTCF across different cell types were downloaded from ENCODE Project [45]. Gene expression from normal tissues was downloaded from GTEx Portal [46]. A detailed description of all used resources and datasets, with an overview and limitations is summarised in Table S2.

### Bioinformatic analyses

For differential expression of ncRNAs in human keratinocytes after p63 knock-down (GSE67382), raw reads were aligned to the human genome (GENCODE19) and gene expression was quantified using STAR package [47] and filtered for the ncRNAs only. The list of annotated ncRNAs was downloaded from the HUGO database. Differential expression analysis was performed using the DEseq2 package [48]. ncRNAs with abs(log2(FC)) > 1 and *P* < 0.05 were considered significantly modulated. Skin-specificity scores were calculated as differences between the *Z*-scores of gene expression in the skin and the maximum value of *Z*-scores in any other tissues. A positive score indicates higher expression in the skin compared to any other tissue, while a negative score indicates at least one tissue with higher expression compared to the skin. Fold-change values and skin-specificity scores are listed in Table S3. scRNA-seq data were analysed using Seurat package [49]. Briefly, cells with feature numbers > 200 and <2000 and <15% of mitochondrial genes were used for the analysis. Dimensionality reductions were performed using UMAP. Cell types were annotated using SingleR package [50]. Pathway enrichment was performed using Metascape [51]. For the analysis of differentiation-specific TF, the list of known human TF was downloaded from The Human Transcription Factors database [52]. Expression values for the TFs were obtained from the GTEx portal or RNA-seq of keratinocyte differentiation in vitro (GSE59827). ChIP-seq and RNA-seq signal tracks were visualised in the UCSC Genome Browser [53]. Heatmaps were generated with pHeatmap package. Volcano plots and correlation density plots were generated using the ggplot2 package [54].

### Statistical analyses

The statistical analyses were performed in GraphPad Prism 9.5.0 (San Diego, USA). For the qPCR analyses, the significance of the differences between the groups was determined by one-way analysis of variance (ANOVA) without adjustments. For the differential gene expression analysis, adjusted *P-*values were calculated using the DEseq2 package in R.

### Supplementary information

Supplementary Figure S1

Supplementary Table S1

Supplementary Table S2

Supplementary Table S3

## Data Availability

All the high-throughput sequencing data used in this study are publicly available and the accession numbers are listed in Materials and Methods.
