# Supplementary material for: Epigenetic priming of an epithelial enhancer by p63 and CTCF controls expression of a skin-restricted gene XP33
Source: Cell Death Discov. 2023 Dec 8;9:446. doi: 10.1038/s41420-023-01716-3 (PMC10709559; doi:10.1038/s41420-023-01716-3)
Supplement: Supplementary file 1 — Supplementary Figure S1 [file 41420_2023_1716_MOESM1_ESM.pdf]

Supplementary Figure S1

A

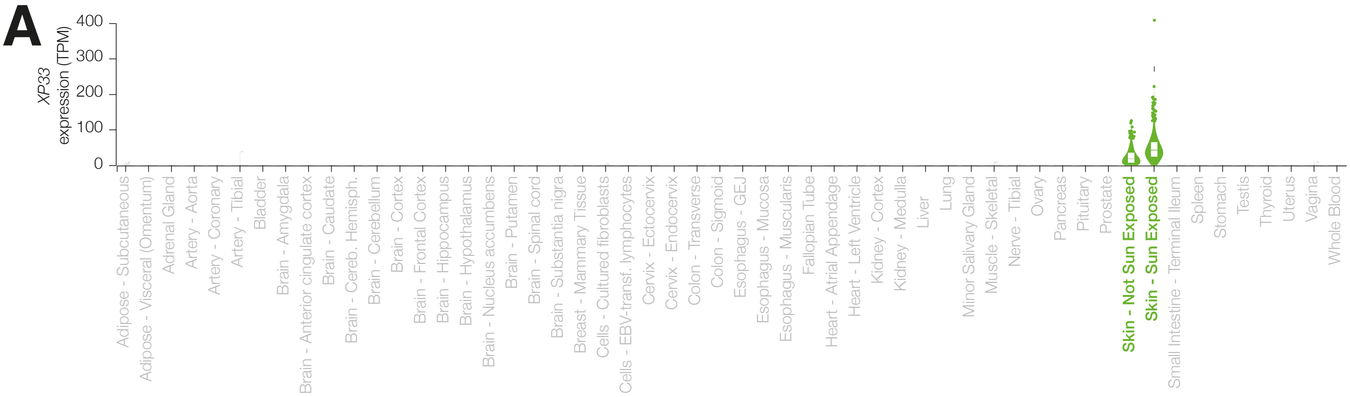

B

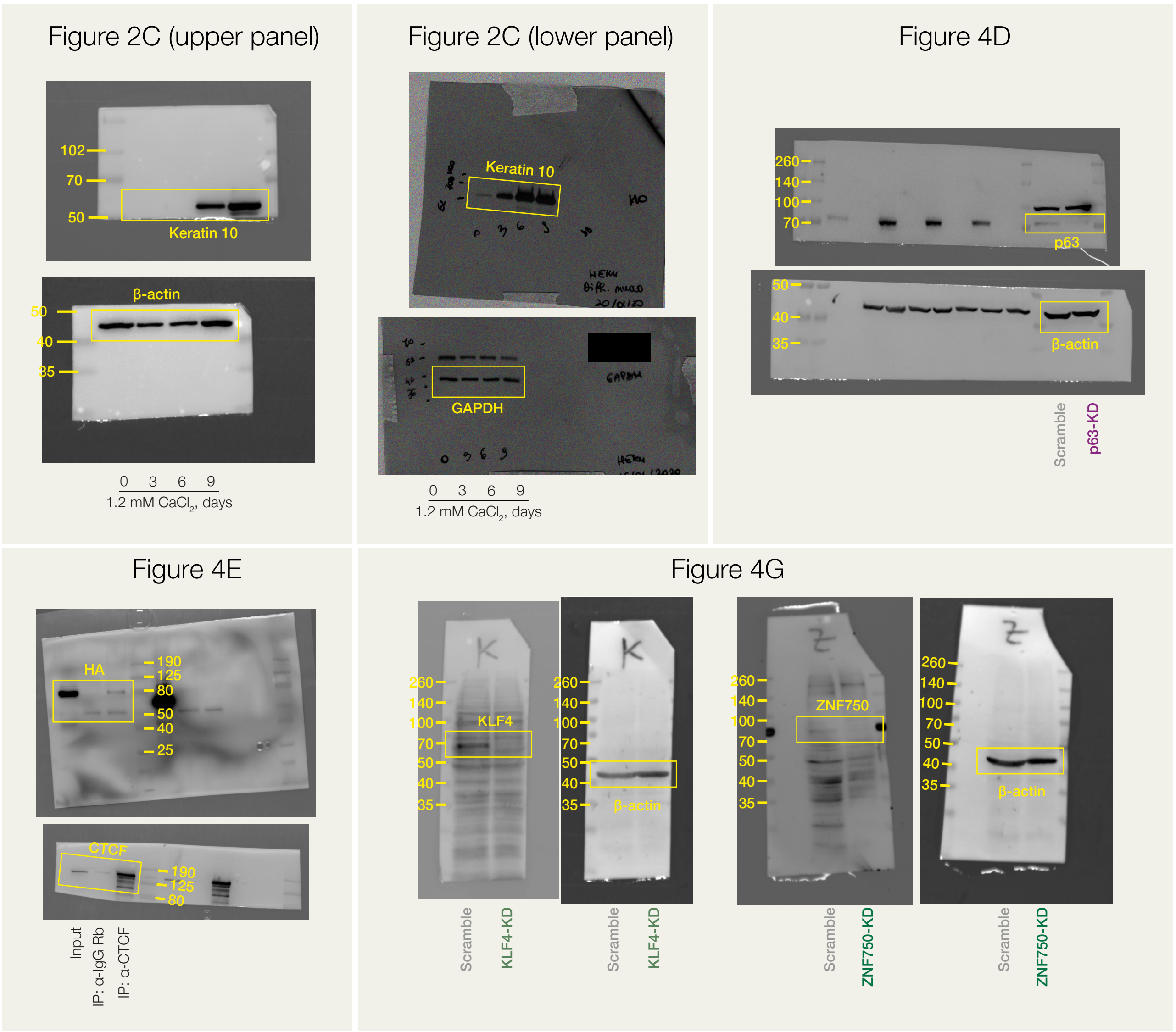

Supplementary Figures legends

Supplementary Figure S1

(A) Violin plots showing XP33 expression across normal tissues from GTEx.

(B) Uncropped version of the western blots from this study.
